# Activation of FcRn Mediates a Primary Resistance Response to Sorafenib in Hepatocellular Carcinoma by Single-Cell RNA Sequencing

**DOI:** 10.3389/fphar.2021.709343

**Published:** 2021-08-06

**Authors:** Xin Guan, Yi Wu, Shuqin Zhang, Zhiyi Liu, Qingjie Fan, Shuai Fang, Sennan Qiao, Fei Sun, Chongyang Liang

**Affiliations:** School of Pharmaceutical Sciences, Jilin University, Changchun, China

**Keywords:** HCC, sorafenib, primary resistance, ScRNA-seq, FcRn

## Abstract

Sorafenib is the first-line therapeutic option for advanced hepatocellular carcinoma (HCC). Many patients exhibit a primary resistance (PR) response after initial treatment. In previous studies, compared to acquired resistance, the mechanism of PR is unclear. The present study aimed to evaluate the response of patient samples to sorafenib by patient-derived xenograft (PDX) models, and the differences at the transcriptome level between the sorafenib PR group and the sorafenib sensitive group were analyzed by single-cell sequencing technology. A specific cell cluster may be differentiated by the liver bud hepatic cells, and the JUN transcription factors in this cell cluster were highly activated. The albumin is secreted by other cell clusters, and the cluster stimulates the FcRn complex receptor to activate the HIF pathway and cell proliferation, resulting in a poor response to sorafenib. These findings are validated by both cell communication analysis and experiments. Thus, the current studies provided a novel approach for the treatment of sorafenib-resistant HCC.

## Introduction

Hepatocellular carcinoma (HCC) is the sixth leading type of cancer and the third most common cause of cancer-related deaths globally (Boucher et al., 2009) The incidence of this fatal disease is rising, and curative treatments, including resection, ablation, or transplantation, are only feasible in patients diagnosed at an early stage (Forner et al., 2016). In the case of patients with advanced HCC, optimal therapeutic options are yet lacking. Sorafenib is the first FDA-approved first-line drug for advanced HCC, which provides limited survival benefits because drug-resistance is an obstacle to extending the overall survival time for HCC patients (Zhu et al., 2017). Several mechanisms, such as crosstalk between PI3K/Akt and JAK-STAT pathways and reprogramming of HIF-regulated glucose metabolism, are involved in the acquired resistance to sorafenib (Trédan et al., 2007). In addition, due to the genetic heterogeneity of HCC, some patients exhibited poor response and resistance to sorafenib, which is termed as primary resistance (PR) (O’Connor et al., 2007). The current understanding of PR is based on the identification of predictive biomarkers; however, the exact mechanism is yet to be elucidated.

In the treatment of lung cancer or breast cancer, tumor biopsy sample sequencing can identify resistance mechanisms and guide next-line therapy, but this is not useful to choose anti-HCC therapeutic agents. Therefore, no researcher has been able to obtain liver cancer tissues from patients who have developed drug resistance after taking sorafenib. Yang et al. developed patient-derived xenograft (PDX) models, in which tumors from HCC patients could be studied after engraftment into immunosuppressed mice (Hu B. et al., 2020). These models were used to identify the indicators of patient response to sorafenib treatment. Herein, we choose some patient tissues that have been surgically removed to establish a PDX model to investigate efficacy and mechanisms of primary resistance. The PDX model retain most of the characteristics of tumor heterogeneity. The tissues from three liver cancer patients were obtained from Crown Bioscience to establish an orthotopic hepatocellular carcinoma model in an attempt to restore the patient’s tumor tissue’s response to sorafenib. According to the *in vivo* efficacy assay, we chose two PR liver cancer tissues for single-cell RNA sequencing and compared the data of the nonefficacy group with the two effective groups, respectively. A special cell cluster was identified to contribute PR to sorafenib. Next, we confirmed the key receptor and ligand in the interaction between this and other cell clusters, which could be used for the development of novel treatment for HCC patients.

## Materials and Methods

### Patient-Derived Tumor Xenograft *in vivo*

PDX models were established in Crown Bioscience (Taicang, China) using tumor fragments subcutaneously transplanted and passaged in female NCG mice. The NCG mice were purchased from Vital River Laboratory Animal Technology Co., Ltd. (Beijing, China). All the animal experiments were performed under sterile conditions in a specific pathogen-free facility, in accordance with the animal welfare laws and the regulations of the Association for Assessment and Accreditation of Laboratory Animal Care (AAALAC). The NCG mice were housed in individually ventilated cages and used at 6 weeks of age. HuPrime® liver cancer xeno-graft models (LI6652, LI6611, and LI6665) and subcutaneous tumors were revived and maintained in NCG mice, respectively, before orthotopic implantation. Identify the heterogeneity of the PDX model by performing immunohistochemistry (IHC) detection on LI6652, LI6611, and LI6665 (Supplementary Figure S1). When the volume reached 500–1,000 mm^3^, the tumors were excised, fragmented into pieces measuring about 2 mm^3^ in diameter, and inoculated into the left lobe of the liver in nude mice. Mice were anesthetized by intraperitoneal injection of 1% pentobarbital sodium. Mice were divided into three groups: in the control group, NCG mice were treated with saline intravenously (i.v.); in the model group, PDX model mice were treated with saline intravenously (i.v.). Above two groups were injected once daily (qd) for 35 days. In the sorafenib group, PDX model mice were treated with 50 mg/kg via oral gavage, once daily for 35 days. The day of implantation was designated as day 0. All the mice euthanized by exposure to carbon dioxide gas; subsequently, the tumors were dissected and measured. All the mice euthanized by exposure to carbon dioxide gas; subsequently, the tumors were dissected and measured. The study was conducted according to the guidelines of the Declaration of Helsinki and approved by the Institutional Animal Care and Use Committee of Jilin University School of Pharmaceutical Science (NO. 2018-0000E24).

### Immunohistochemistry

Specimens were fixed in 10% formalin and submitted to embedding in paraffin according to standard histological procedures. Anti-EGFR (1:400, Abcam, Cat# ab52894) and anti-c-Met (1:400, Abcam, Cat# ab51067) were used as primary antibodies. UltraVision Quanto Detection System HRP DAB (Thermo,Cat#TL060QHD) was used for endogenous peroxide blocking and color development. The hematoxylin solution was used for counterstaining. Finally, the finished sections were observed under microscopy.

### Single-Cell Dissociation

The tissues were removed surgically and placed in MACS Tissue Storage Solution (Miltenyi Biotec, Cat# 130100008) until further processing. Briefly, the samples were first washed with phosphate-buffered saline (PBS), minced into small pieces (approximately 1 mm^3^) on ice, and digested with 200 U/mL collagenase I (Worthington, Cat# LS004197) and 200 U/mL DNase I (Worthington, Cat# LS002147) for 45 min at 37°C, with agitation. Subsequently, the samples were sieved through a 70-µm cell strainer and harvested by centrifugation at 300 rpm for 5 min at room temperature. After washing with PBS containing 0.04% bovine serum albumin (BSA), the cell pellets were re-suspended in PBS containing 0.04% BSA and re-filtered through a 35-μm cell strainer. The viability of the dissociated single cells was assessed using Calcein-AM (Thermo Fisher Scientific, Cat# c3099) and Draq7 (BD Biosciences, Cat# 564904).

### Single-Cell RNA Sequencing

BD Rhapsody system was used to capture the transcriptomic information of the single cells. Single-cell capture was achieved by the random distribution of a single-cell suspension across >200,000 microwells through a limited dilution approach. Beads with oligonucleotide barcodes were added to saturation, such that one bead was paired with a cell in a microwell. Cell-lysis buffer was added so that poly-adenylated RNA molecules hybridized to the beads. Then, the beads were collected for reverse transcription. After synthesis, each cDNA molecule was tagged on the 5′-end (that is, the 3′-end of an mRNA transcript) with a unique molecular identifier (UMI) and cell label indicating its cell of origin. Whole transcriptome libraries were prepared using the BD Rhapsody single-cell whole-transcriptome amplification workflow. Briefly, second-strand cDNA was synthesized, followed by ligation of the WTA adaptor for universal amplification. The adaptor-ligated cDNA products were amplified by 18 PCR cycles. Sequencing libraries were prepared using random priming PCR of the whole-transcriptome amplification products to enrich the 3′-end of the transcripts linked with the cell label and UMI. The sequencing libraries were quantified using a High Sensitivity DNA chip (Agilent) on a Bioanalyzer 2,200 and Qubit High Sensitivity DNA assay (Thermo Fisher Scientific). The library for each sample was sequenced on an Illumina sequencer (Illumina) using a 150 bp paired-end run.

### Single-Cell RNA Statistical Analysis

scRNA-seq data were analyzed by NovelBio Bio-Pharm Technology Co., Ltd. (Shanghai, China) on the NovelBrain Cloud Analysis Platform. We applied fastp with the default parameter to filter the adaptor sequence and remove the low-quality reads and achieve clean data (Chen et al., 2018). UMI tools were applied for single-cell transcriptome analysis to identify the cell barcode whitelist (Smith et al., 2016). The UMI-based clean data were mapped to the human genome (Ensemble version 91) utilizing STAR mapping with customized parameters from the UMI tools standard pipeline to obtain the UMI counts of each sample (Dobin et al., 2013). The cells containing >200 expressed genes and mitochondria UMI rate <80% passed the cell quality filtering; the mitochondrial genes were removed in the expression table. Seurat package (version: 2.3.4, https://satijalab.org/seurat/) was used for cell normalization and regression based on the expression table according to the UMI counts of each sample and the mitochondria rate to obtain scaled data. PCA was constructed based on the scaled data with top 2000 high variable genes, and top 10 principals were used for tSNE construction and UMAP construction.

The graph-based cluster method (resolution = 0.8) was used to acquire the unsupervised cell cluster data based on the PCA’s top 10 principals. Then, the marker genes were estimated by the FindAllMarkers function using the Wilcox rank-sum test algorithm under the following criteria: 1. lnFC>0.25; 2. *p-*value < 0.05; 3. min. pct >0.1. In order to identify the cell type, the clusters of the same cell type were selected for re-tSNE analysis, graph-based clustering, and marker analysis.

### Pseudotime Analysis

We applied the Single-Cell Trajectory analysis utilizing Monocle2 (http://cole-trapnell-lab.github.io/monocle-release) using DDR-Tree and default parameters. Before Monocle analysis, the marker genes were selected from Seurat clustering data and raw expression counts of the cells that passed filtering. Based on the pseudotime analysis, branch expression analysis modeling (BEAM) was applied to branch fate gene analysis.

### Cell Communication Analysis

To enable a systematic analysis of cell-cell communication molecules, we applied cell communication analysis based on the CellPhoneDB, a public repository of ligands, receptors, and their interactions (Vento-Tormo et al., 2018). Membrane, secreted, and peripheral proteins of the cluster of different time points were annotated. Cell communication (*p*-value<0.05) was assessed based on the interaction, and a normalized cell matrix was achieved by Seurat normalization.

### SCENIC Analysis

We applied the single-cell regulatory network inference and clustering (pySCENIC, v0.9.5) workflow to assess the transcription factor regulation strength, using the 20-thousand motifs database for RcisTarget and GRNboost (Aibar et al., 2017).

### QuSAGE Analysis (Gene Enrichment Analysis)

The relative activation of a given gene set, such as pathway activation, was characterized using QuSAGE (2.16.1) analysis (Yaari et al., 2013).

### Differential Gene Expression Analysis

To identify the differentially expressed genes among samples, the function FindMarkers with Wilcox rank-sum test algorithm were used under the following criteria: 1. lnFC>0.25; 2. *p-*value < 0.05; 3. min. pct>0.1.

### Co-Regulated Gene Analysis

To identify the gene co-regulation network, find gene modules function of monocle3 was utilized with the default parameters (Cao et al., 2019).

### Cell Culture

The human HCC line Huh-7 (WT) (Serial, TCHu182) was purchased from the National Collection of Authenticated Cell Cultures and cultured in DMEM (BI, Cat# 061055571A) with 10% (vol/vol) fetal bovine serum (FBS, Gibco, Cat# 12483020). The human HCC line sorafenib resistance Huh-7(SR) (XC509) was purchased from MEIXUAN Biological Science and Technology Ltd. (Shanghai, China), and cultured in RPMI-1640 medium (BI, Cat# 011001A) with 10% (vol/vol) FBS mixed with sorafenib (2 μg/ml final concentration). The cells were cultured at 37°C in a 5% CO_2_ incubator.

### Flow Cytometric Analysis

Huh-7 (WT) and Huh-7 (SR) cells were seeded into the 6-well plate (2×10^5^ cells/well). After 18 h, the cells were treated with serum starvation for 6 h and 10% (vol/vol) ALB (Roche, Cat# 10735078001) was added for 6 h after serum starvation. Then, the cells were harvested, washed twice with PBS, and incubated with purified mouse monoclonal FcRn antibody (Santa Cruz, Cat# sc-271745, RRID: AB_10707665) at room temperature for 1 h. Finally, the cells were analyzed using integrated Cytomics FC 500 (Beckman Coulter) cxp 2.1 software.

### Immunofluorescence Analysis

Immunofluorescence was accomplished by growing cells on glass coverslips. The cells were treated with serum starvation for 6 h, and 10% (vol/vol) ALB was added for 6 h after serum starvation. Then, the cells were fixed with 4% paraformaldehyde in PBS for 20 min and permeabilized with 0.1% Triton X-100 for 10 min at room temperature, followed by incubation with primary antibody (Abcam, Cat# ab228975) for 1 h at room temperature and relevant secondary Alexa Fluor antibodies at 1:1,000 for 1 h. Live-cell images were captured after the cell nuclei were stained using Hoechst 33342 (Thermo Fisher Scientific Cat# 639 RRID: AB_2651135). Cells were imaged using OLYMPUS Fluorescence Microscope IX71.

### Construction of RNA Sequencing Libraries and Sequencing

Total RNA was extracted from the samples using TRIzol reagent (Invitrogen, Cat# 15596026). The quality was evaluated on Agilent 2,200, and the RNA samples were preserved at −80°C. The RNA with RNA integrity number (RIN) > 7 is optimal. The cDNA libraries were constructed for each RNA sample using the TruSeq Stranded mRNA Library Prep Kit (Illumina), according to the manufacturer’s instructions. Typically, the protocol consists of the following steps: Poly-A containing mRNA was purified from 1 mg total RNA using oligo (dT) magnetic beads and fragmented into 200–500 bp using divalent cations at 94°C for 5 min. The cleaved RNA fragments were used for first- and second-strand cDNA synthesis. dUTP mix was used for second-strand cDNA synthesis, which allows for the removal of the second strand. Then, the cDNA fragments were end-repaired, A-tailed, and ligated with indexed adapters. The ligated cDNA products were purified and treated with uracil DNA glycosylase to remove the second-strand cDNA. The purified first-strand cDNA was enriched by PCR to construct the cDNA libraries. The quality was assessed using Agilent 2,200, and the libraries were sequenced by HiSeq X (Illumina) on a 150 bp paired-end run.

### RNA Sequencing Mapping

Before mapping, clean reads were obtained from raw reads by removing the adaptor sequences and low-quality reads. The clean reads were then aligned to the human genome (GRCh38, NCBI) using the Hisat2 (Kim et al., 2015). HTseq was used to obtain gene counts, and the RPKM method was used to determine the gene expression (Anders et al., 2015).

### Gene Ontology (GO) Analysis

GO analysis elucidated the biological implications of the differentially expressed genes in the experiment (Ashburner et al., 2011). We downloaded the GO annotations from NCBI (http://www.ncbi.nlm.nih.gov/), UniProt (http://www.uniprot.org/), and the GO (http://www.geneontology.org/) Databases. Fisher’s exact test was applied to identify the GO categories (*p*-value < 0.05).

### Pathway Analysis

Pathway analysis was used to identify the significant pathway of the differentially expressed genes according to the KEGG database. Fisher’s exact test was applied to select the significant pathway, and the threshold of significance was defined by *p*-value <0.05 (Draghici et al., 2007).

### Huh7 FcRn Knockout Cell Line by CRISPR/Cas9

The FcRn knockout from the Huh7 WT cell line by CRISPR/Cas9 system (SpCas9). Transform SpCas9 plasmid into *E. coli* cells. The *E. coli* colonies were genotyped by PCR for deletion of a 270 bp amplicon containing the gRNA spacer and U6 promoter. Purified CRISPR plasmids were transfected into Huh7 cells and selected with Puromycin (1 μg/ml) for 5 days. Single cell colony expansion was applied after CRISPR transfection into Huh7 to obtain monogenic knockout clones.

### Western Blot

Western blotting was performed according to the protocol of Bio-Rad semi-dry transfer using the Bio-Rad Transfer Cell System. Podocytes were harvested by scraping into RIPA buffer and protein concentrations were determined by bicinchoninic acid assay (Thermo Fisher Scientific Cat# 23225). Anti-FcRn (1:1,000, Santa Cruz, Cat# 271745) was used as primary antibody in detection. Goat anti-mouse IgG-HRP (1:5,000, Santa Cruz, Cat# 2005)was used as secondary. The antibody complexes were detected using enhanced chemiluminescence (Tanon, Cat# 180501) and Western blot images were captured using a photodocumen-tation system (Tanon-4500).

### Cell Viability Assays

Cell viability was monitored with CCK8 kit (MCE, Cat# HYK0301) following the producer’s suggestions. The cells (8,000 cells/well) were cultivated in 96-well plates with three replicate wells. 10 ul CCK-8 solution was added to each well, incubated for 2 h, and then assayed using a microplate reader with a wavelength of 450 nm.

## Results

### PDX Model Evaluated the Therapeutic Effect of Sorafenib

**FIGURE 1:**
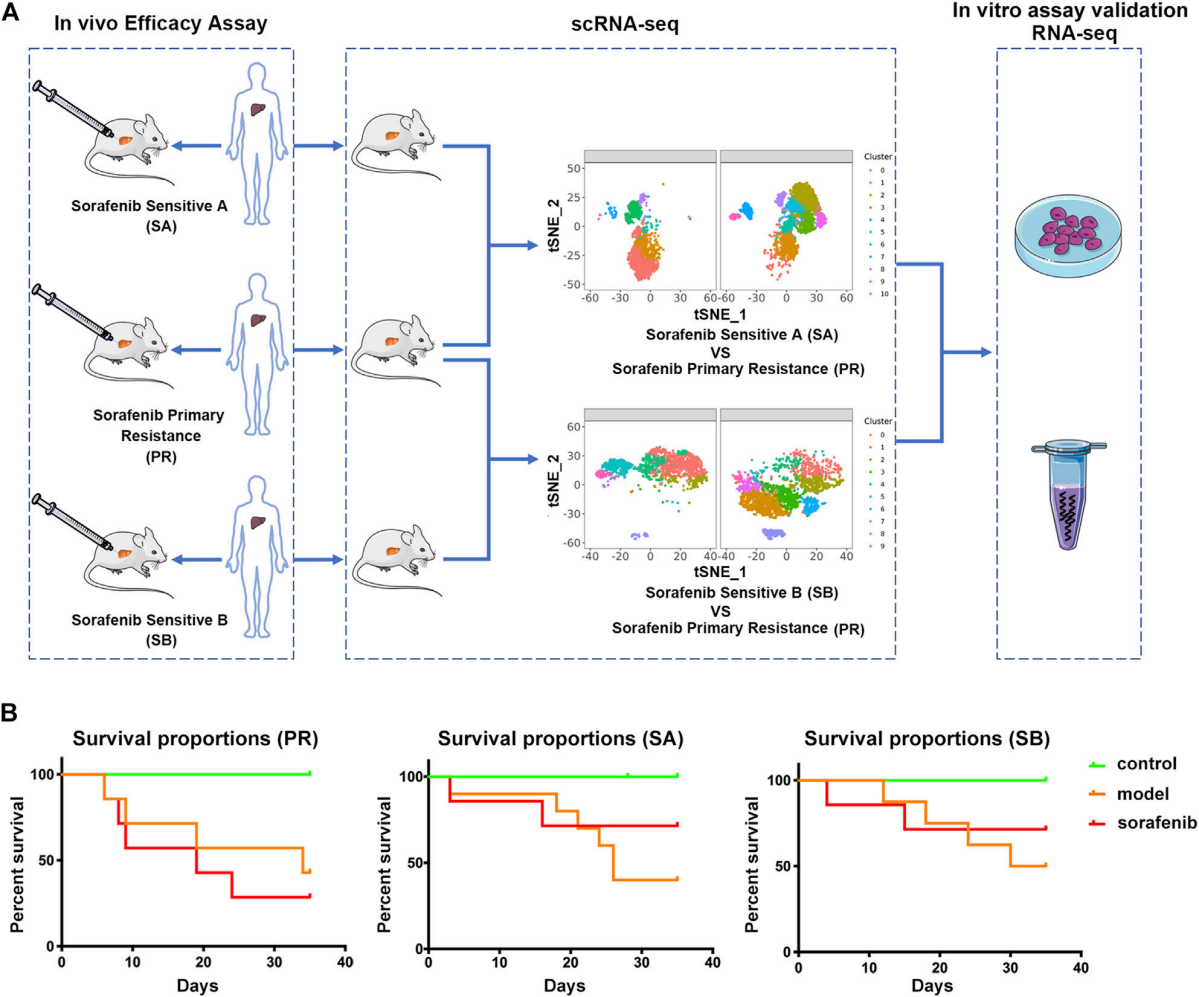
Characterizing sorafenib primary resistance cell in HCC by Single-Cell RNA-Seq. **(A)** Workflow of the sample preparation, sequencing and bioinformatic analysis **(B)** Statistics of survival rate of three groups of PDX model mice. Tumor tissues of three HCC patients were grown as orthotopic xenografts. In the control group, NCG mice were treated with saline intravenously (i.v.); in the model group, PDX model mice were treated with saline intravenously (i.v.). Above two groups were injected once daily (qd) for 35 days. In the sorafenib group, PDX model mice were treated with 50 mg/kg via oral gavage, once daily for 35 days. All PDX mice were sacrificed on the 35th day.

In the present study, we explored the mechanism of patients’ PR to sorafenib in the clinic. The PDX model was used to evaluate the drug effect, then compared the difference between the effective group of sorafenib and the PR group, and finally verified the results (Figure 1A). We inoculated three liver cancer tissues from different patients into the liver of mice to establish PDX models. Considering the heterogeneity of liver cancer and the microenvironment that mimics the real liver cancer tissue, we adopted the tumor-in-situ model, and the survival rate was used as the first indicator to evaluate the therapeutic effect of sorafenib. Next, we selected a group of PDX models that did not respond to sorafenib treatment, with a survival rate of 28.57%; this was defined as the sorafenib primary resistance (PR) group. The other two groups of PDX models with survival rates of 71.42% were defined as sorafenib sensitive A (SA) and sorafenib sensitive B (SB) control groups and compared to the PR group, respectively (Figure 1B). Thus, the corresponding target cell cluster was identified, and the differences between the cell clusters were explained comprehensively.

### scRNA-Seq Analysis of PR Cell Cluster

**FIGURE 2:**
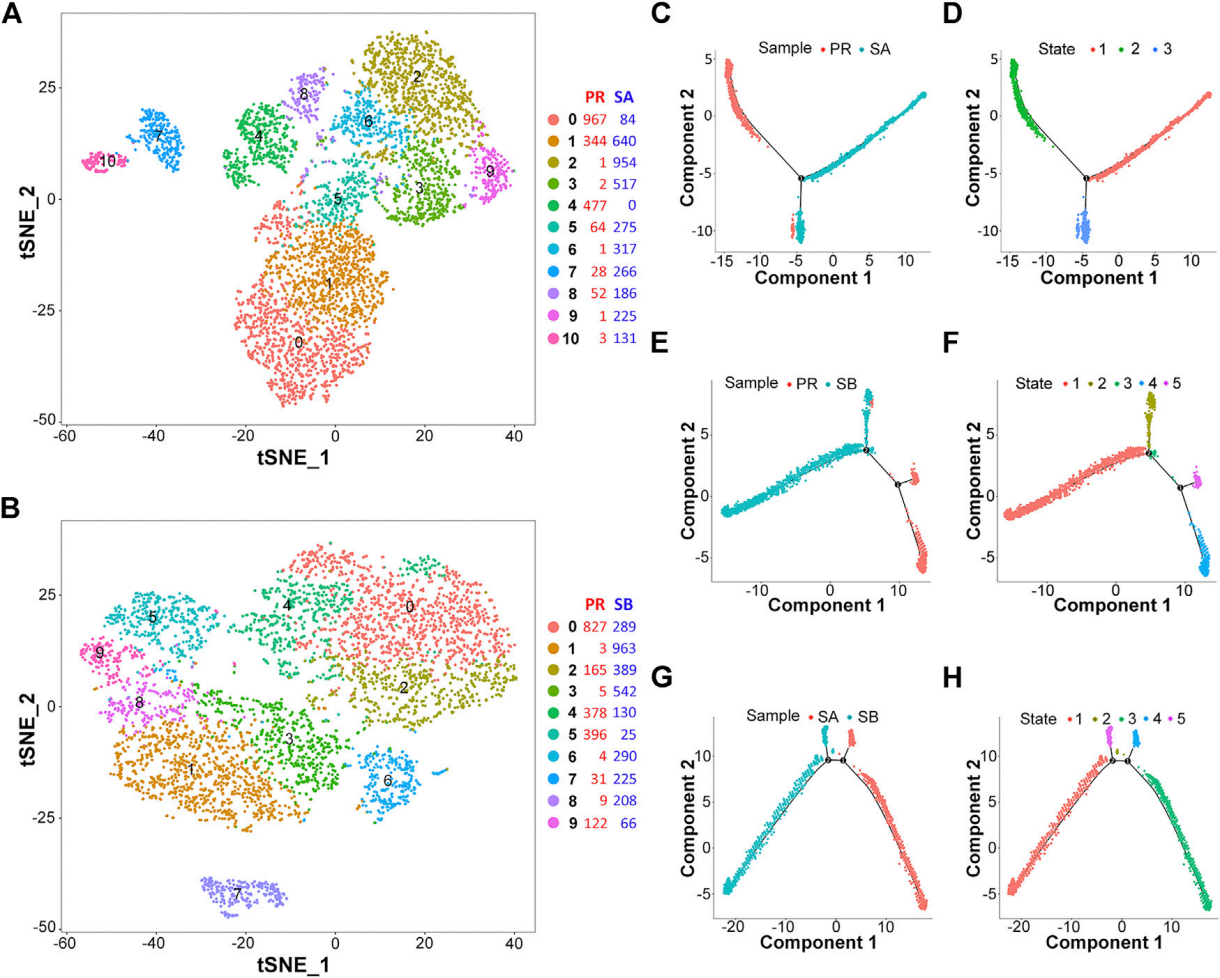
Differentiation heterogeneity of HCC cells. **(A)** Two-dimensional tSNE plot depicting 5,535 single cells of PR-SA group, each classified into the 11 clusters shown with distinct colors. **(B)** Two-dimensional tSNE plot depicting 5,067 single cells of PR-SB group, each classified into the 10 clusters shown with distinct colors. **(C–H)** Pseudotime analysis of cell clusters in PR-SA/PR-SB/SA-SB groups showed a different differentiation processes.

In order to evaluate the heterogeneity of liver cancer cells, we set PR and SA into one group, and PR and SB into another group, followed by scRNA-seq analysis separately. First, based on the gene expression data in each cell, we used a clustering algorithm to analyze the subpopulation of cluster cells. Also, t-SNE analysis was used to visualize the cell cluster data (Figures 2A,B, Supplementary Figures S1A,B). Then, the cell types were assessed based on the differences in gene expression between various cell clusters. Since our samples were obtained from liver cancer tissues, the cell types were mainly liver cancer cells or immune cells. While analyzing and labeling the cell cluster types, *p*-value was set at <0.1, and the number of marker genes was >10 to distinguish the cell types or the differentiation states of the same type of cells. As shown in Supplementary Table S1, the PR-SA comparison data revealed that clusters 0, 2, 3, 4, 6, and 9 might be tumor cells derived from liver bud hepatic cells, and cluster seven is an immune cell cluster based on exhausted CD4^+^ T cells. In the PR-SA comparison data, cluster 0 is mainly derived from liver bud hepatic cells and regulatory T cells. Cluster 1 is mainly derived from liver bud hepatic cells, regulatory T cells, and exhausted CD8^+^ T cells. Cluster two constitutes exhausted CD8^+^ T cells and regulatory T cells. Clusters 3, 5, and 6 are derived from liver bud hepatic cells and regulatory T cells, while cluster 7 mainly consists of regulatory T cells. This phenomenon shows that tumor cell clusters may be differentiated from the same type of cells, but due to the differences in their gene expression, the development and differentiation of cell clusters vary, which ultimately leads to diversified functions of cell clusters.

Intriguingly, we used the pseudotime analysis technology to analyze the cell lineage and the sequence of cell-to-cell transformation and succession based on the changes in gene expression of different cell subgroups. The synthesis of t-SNE and pseudotime analysis results in the PR-SA group revealed 0, 1, 4, 5, and 8 clusters of cells, because a large proportion of these groups of cells is distributed in the second state, i.e., (Supplementary Figures S1C,E), the PR state. Cluster four of cells is rather specific because all cells are in the PR state (Figures 2C,D). Also, in the PR-SB group, the major proportion of 0, 2, 4, 5, and 9 clusters of cells are distributed in the PR state (Figures 2E,F, Supplementary Figures S1F–H). Next, we performed a pseudotime analysis of the SA-SB group, and the results showed that the trajectory of transformation between the two groups of sorafenib sensitive cells differed markedly (Figures 2G,H). This finding also showed that SA and SB substantiate the mechanism of PR cell cluster from different aspects.

### JUN Regulates HIF Pathway to Antagonize Sorafenib in Liver Bud Hepatic Cells

Several studies reported that sorafenib treatment often causes hypoxia in tumor tissues, and the activation of HIF pathway facilitates tumor cell growth under hypoxic conditions (Wilson et al., 2014) (Zhao et al., 2014) (Piret et al., 2006).

**FIGURE 3:**
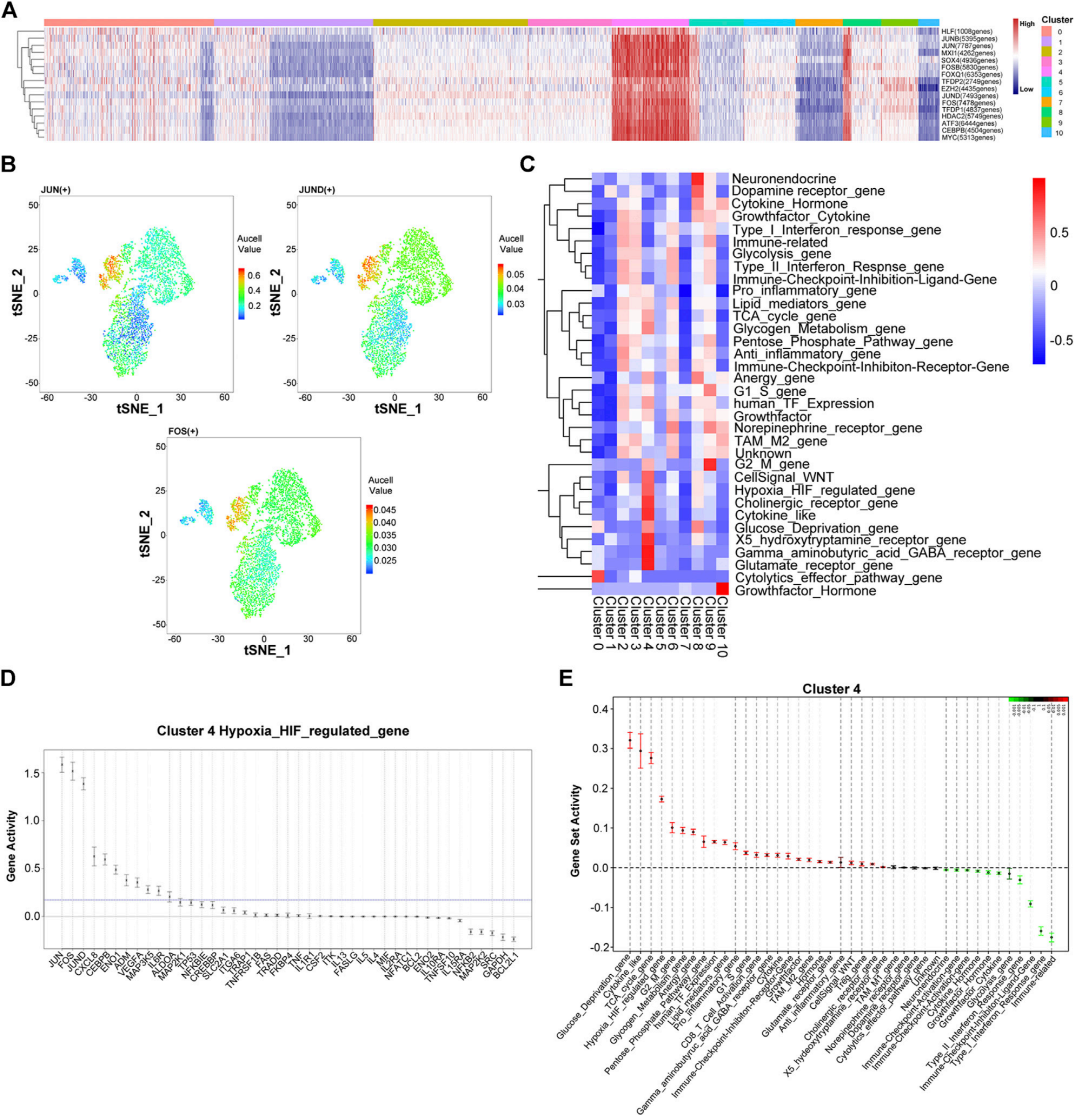
Heterogeneity of gene expression and metabolic pathways in sorafenib poor response cell in the PR-SA group. **(A)** Heatmap showed activation intensity of the genes in tSNE-assigned cell clusters. Red means genes were activated, blue means genes were inhibited. **(B)** Transcription factor JUN, JUND, FOS activation degree in the PR-SA group. The color represents the degree of gene activation. **(C)** QuSAQE analysis of metabolic activities. Red means metabolic was activated, blue means metabolic was inhibited. **(D)** Hypoxia metabolism regulated gene activity strength in cluster 4. **(E)** Gene set activity strength in cluster 4. Red means gene set was activated, blue means gene set was inhibited.

Next, we utilized a single-cell regulatory network inference and clustering (SCENIC) to identify and characterize active gene-regulatory networks across the PR-SA group. As shown in Figure 3A, the activation of transcription factors was observed between cluster four and other clusters. The activation intensity of HIF pathway regulatory genes, *JUN*, *FOS*, and *JUND*, was significantly higher than that of the other cluster of cells (Figure 3B). *JUN, FOS* and *JUND* are all related to the proliferation, apoptosis, and differentiation of HCC (Malz et al., 2012). The core transcription factor intensity of some cells in cluster eight was similar to that of cluster 4. Since clusters four and eight in tSNE are adjacent, some cells may have overlapping characteristics. The Quantitative Set Analysis for Gene Expression (QuSAGE) analysis revealed that the activation degree of the signaling motif of cluster four differed significantly from that of other clusters. The upregulation of the TCA cycle and G2/M and G1/S signaling pathways indicated that cluster four could be differentiated and reproduced under the pressure of sorafenib. The upregulation of the glucose deprivation, hypoxia HIF-regulated, glycogen metabolism, and pentose phosphate pathways indicated that cluster four had undergone HIF pathway metabolism under hypoxia (Figures 3C,E) (Wilson et al., 2014; Chen and Lou, 2017). Similar to the results of SCENIC, we found that *JUN, FOS,* and *JUND* genes were activated in the HIF regulatory pathway (Figure 3D).

This analysis was repeated for the PR-SB group. The SCENIC analysis revealed that the activation of transcription factors in clusters five and cluster nine of cells was significantly different from the other cell clusters (Supplementary Figure S2). The activation intensity of the HIF pathway regulatory gene, *JUNB*, was also significantly higher than that of other clusters. According to QuSAGE analysis, the activated signaling pathways of cells in cluster five and cluster nine are identical, except that the activation degree of cluster nine is slightly weaker than that of cluster 5. The upregulation of the TCA cycle indicated that the cluster five cells undergo differentiation and proliferation. The upregulation of glycolysis, pentose phosphate pathway, hypoxia HIF-regulated, and glucose deprivation indicated that the cells of cluster five undergo HIF pathway metabolism. The upregulation of G2M, TCA cycle, and G1S activation indicated that cluster nine cells undergo cell differentiation and proliferation. The upregulated glycolysis, pentose phosphate pathway, hypoxia HIF-regulated, and glucose deprivation indicated that the cells of cluster nine undergo HIF pathway metabolism.

### Albumin-FcRn Is the Core Communication Between PR and Other Cell Clusters

**FIGURE 4:**
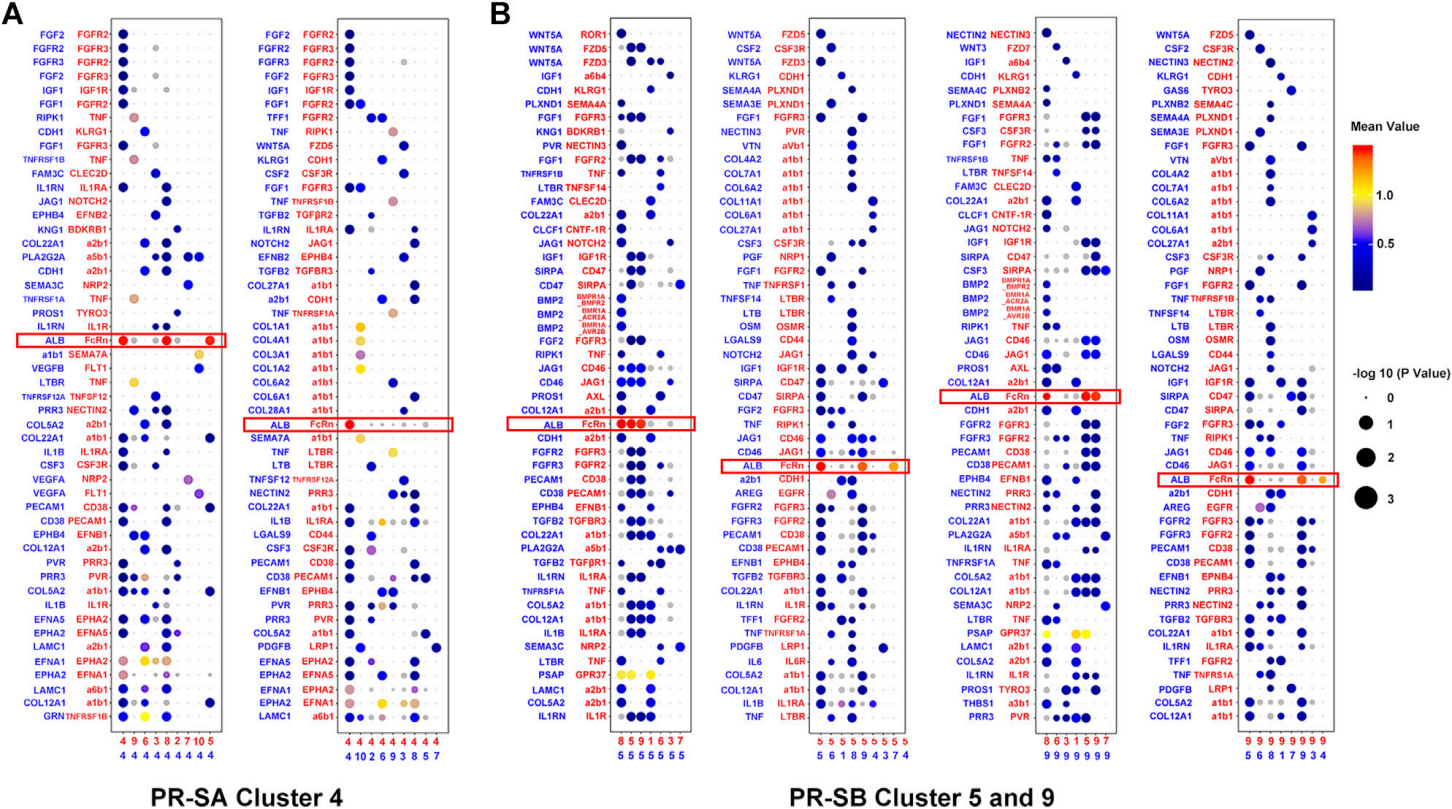
Cell-cell communication network between PR cell clusters and other cell clusters. **(A)** Bubble plots showed the interaction strength of ligand-receptor pairs of cluster four in the PR-SA group. **(B)** Bubble plots showed the interaction strength of ligand-receptor pairs of cluster five and nine in the PR-SB group. Red represented receptors and blue represented ligands. The color and area of the dots represent the intensity of the interaction.

In order to understand the characteristics of the key cell cluster of PR, we used Cell-PhoneDB (a public repository of ligands, receptors, and their interactions) for cell communication analysis. In the PR-SB group, we observed that clusters five and nine of cells interact *via* ALB and FcRn, and both stimulate cluster eight by ALB (Figure 4B). The communication between other cell clusters is isolated. Since clusters five and nine are adjacent in the t-SNE analysis, the transcription factors, *JUNB*, *FOXQ1*, and *TRIM28*, are activated in these two clusters of cells, and the process of cell differentiation was similar in pseudotime analysis (Supplementary Figures S1F,G). Therefore, we speculated that clusters five and nine indicate that ALB and FcRn induce two differentiated states of the cells. According to the data of modules 7 and 22, we found that the cells of cluster 9 may induce the cells of cluster five to dedifferentiate towards proliferation, as shown by co-regulated gene analysis. When cells of clusters five and nine induce each other to the final state, it may be the same state as cluster four cells in the PR-SA group (Supplementary Figure S3B, Table S4, 5). Since cluster eight is adjacent to clusters five and nine in the t-SNE analysis, we speculated that clusters five and nine also induce cluster eight to differentiate into the PR.

In the PR-SA group, we observed that cluster four interacted highly with itself and clusters five and eight by ALB and FcRn (Figure 4A). We observed that cluster four is in the PR state, as assessed by pseudotime analysis. The differentiation status of clusters five and eight is similar, and both differentiated towards PR status (Supplementary Figures S1C,D). Cluster four is adjacent to clusters five and eight in the t-SNE analysis. According to the data of modules 44 and 55, we found that cluster 4 may induce clusters five and eight to dedifferentiate in the direction of strong proliferation, i.e., PR by co-regulated gene analysis (Supplementary Figure S3A, Table S2, 3).

### FcRn Activation by ALB May Trigger PR

Since the samples from single-cell sequencing cannot be used for verification experiments, we input a query in the CCLE (Cancer Cell Line Encyclopedia) database, and the huh-7 sorafenib resistance (SR) and huh-7 wild-type (WT) cell lines with relatively high *fcgrt* gene expression were selected for substantiation experiments. In order to verify the role of the interaction between ALB and FcRn in PR, we divided HUH-7 SR and huh-7 WT cell lines into four experimental groups: serum starvation for 6 h and 10% BSA supplementation after 6 h of starvation.

**FIGURE 5:**
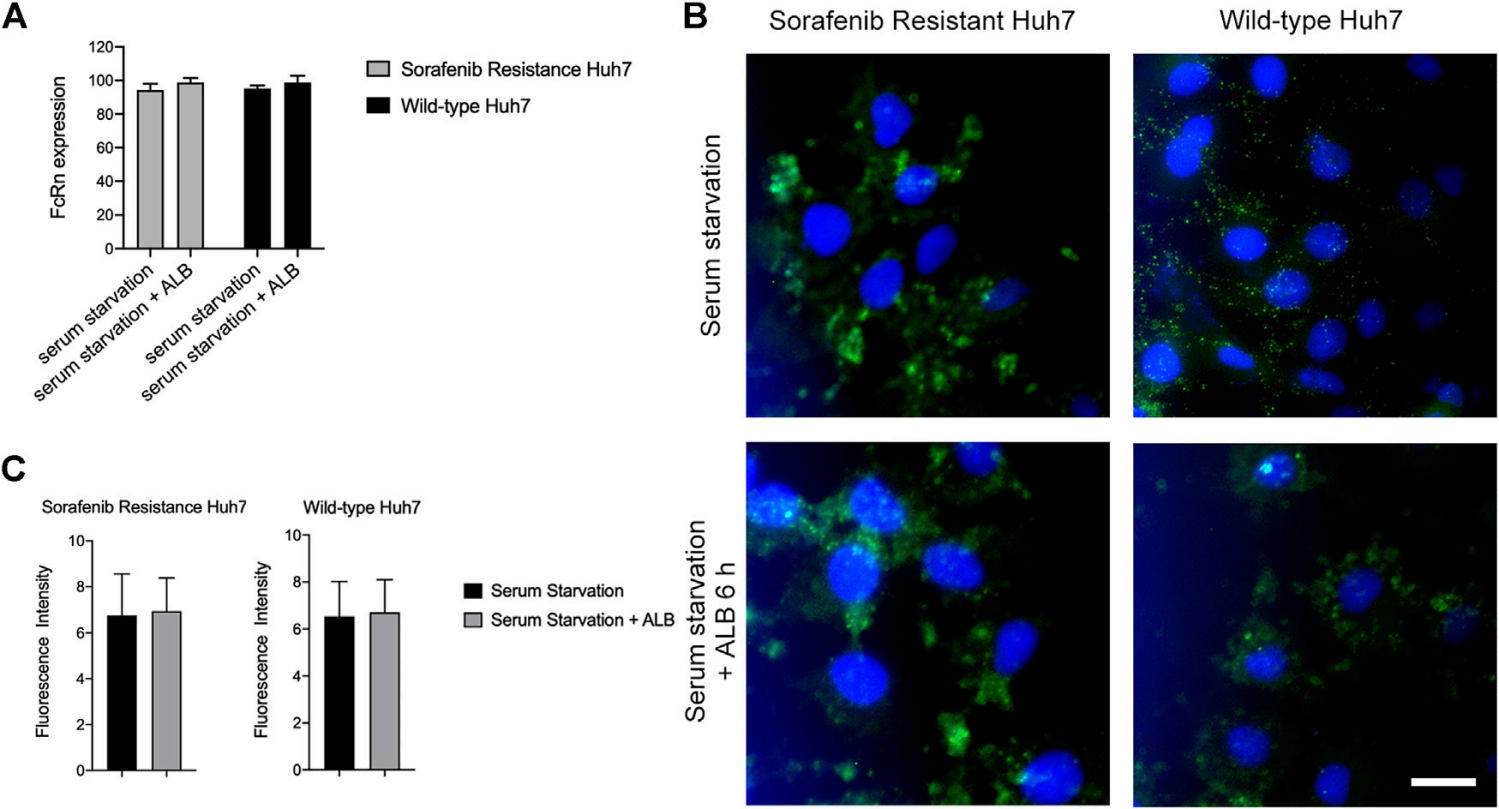
The expression of FcRn in huh7 (SR) and huh7 (WT). **(A)** FcRn expression of Huh7 (SR) and Huh7 (WT) cells in serum starvation 6 h and adding ALB after serum starvation 6 h, as determined by flow cytometry. **(B-C)** FcRn expression (green) of Huh7 (SR) and Huh7 (WT) cells in serum starvation 6 h and adding ALB after serum starvation 6 h, as determined by Immunofluorescence. Cell nuclei were dyed by Hoechst (blue). Scale bar: 10 μm.

We evaluated the differences in the expression of FcRn on cells in various experimental groups via flow cytometry and immunofluorescence. Based on the experimental results, we discovered that cells were stimulated with ALB after serum starvation and the expression of the FcRn receptor increased compared to the serum starvation group (Figure 5A). Immunofluorescence microscopy confirmed the flow cytometry results (Figures 5B,C). Therefore, we inferred that the root cause of sorafenib resistance in cells is not the level of FcRn expression in cells but the genetic differences in the cells.

**FIGURE 6:**
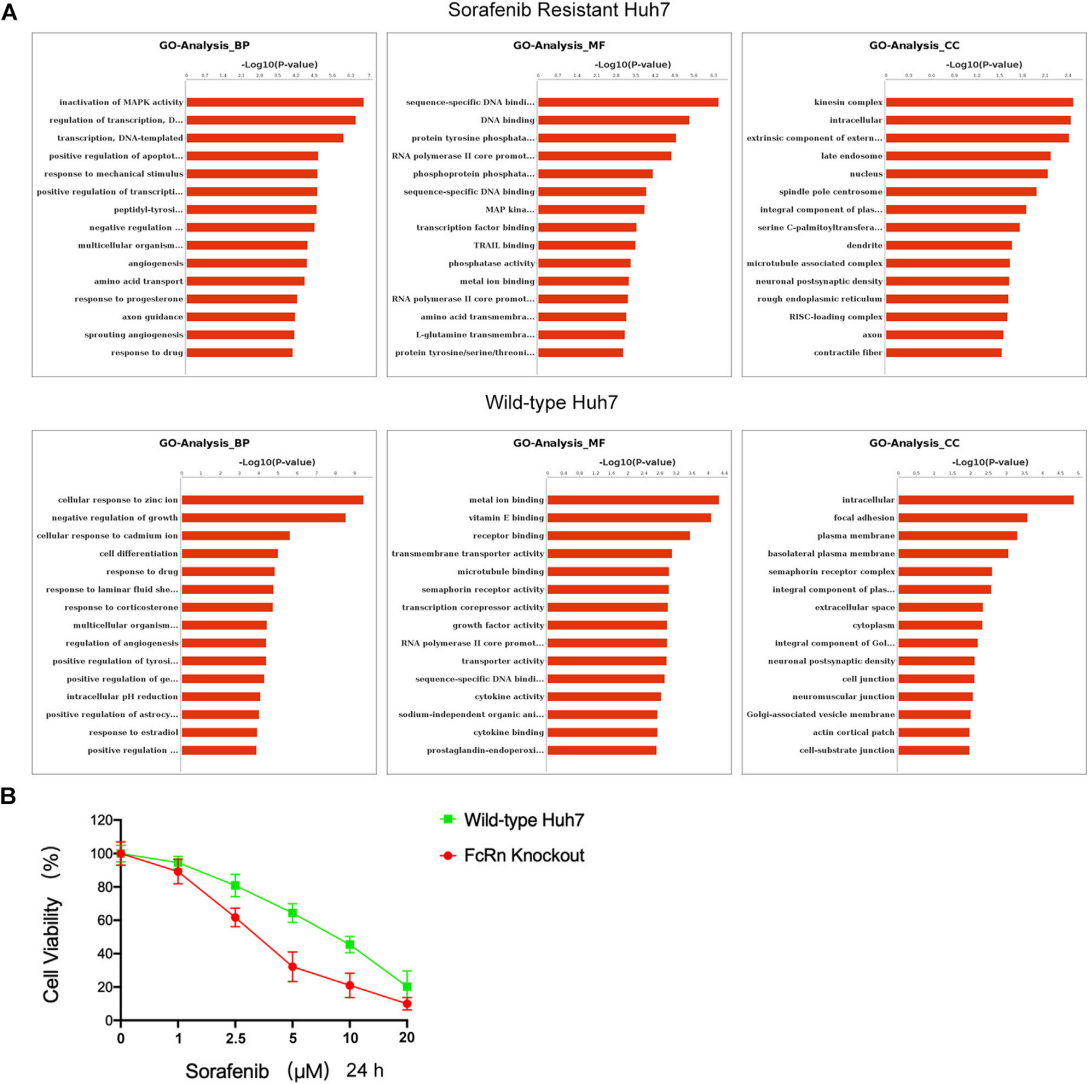
ALB-FcRn interaction caused the proliferation of sorafenib-resistant cells. **(A)** GO analysis of Huh7 (SR) and Huh7 (WT) cells which were added ALB after serum starvation 6 h by RNA-seq. **(B)** Huh7(WT) and Huh7(KO) were cultured with increasing doses of sorafenib (0–20 μM) for 24 h incubation. The percentage of surviving cells in relation to the controls was determined by CCK8.

Subsequently, RNA-seq analysis was performed. The GO analysis indicated that the HUH-7 SR cell line and the HUH-7 WT in biological process (BP), molecular function (MF), and cellular components (CC) are different (Figure 6A). The main differences in BP between the serum starvation group and the ALB stimulation group of the HUH-7 SR cell line regulate the MAPK activity, DNA template transcription, and apoptosis. The main differences in MF are observed in DNA binding, transcription factor binding, and tyrosine/serine/threonine phosphatase activity. The main differences in CC are intracellular function, late endosome, and nucleus. The main differences in BP between the serum starvation group and the ALB stimulation group of the HUH-7 WT cell line are observed in the cell response to ions, cell differentiation, and drug response. The main differences in MF focus on metal ion binding, vitamin E binding, and receptor binding. The main differences in CC are detected in the intracellular function, plasma membrane, and cytoplasm regions. It was concluded that HUH-7 sorafenib-resistant cell line showed cell proliferation and differentiation after ALB stimulation, and these processes are related to the HIF pathway (Sang et al., 2003; Piret et al., 2006; Mole et al., 2009; Gammella et al., 2010; Malz et al., 2012; Hammami et al., 2018; Koeppen et al., 2018; Hu H. et al., 2020). While normal HUH-7 cell lines did not present a similar state.

To further confirm the results of sc-RNA sequencing, we were constructed Huh7 FcRn-knockout (KO) cell line according to Howard et al.’s instructions (Larsen et al., 2020). The FcRn expression were verified by western blot analysis (Supplementary Figure S5). In addition, the effect of sorafenib on the viability of Huh7 FcRn (WT) and Huh7 FcRn (KO) were evaluated by CCK8 assay for 24 h. The dose dependent manner was shown in Figure 6B. Compared with Huh7 FcRn (WT), Huh7 FcRn (KO) shows lower cell viability. This shows that the expression of FcRn may affect the resistance of sorafenib.

## Discussion

The single-cell sequencing data in this study showed that there might be an over-activated cell cluster of the *JUN* gene in the liver tumor tissues of patients who have not used sorafenib. The HIF pathway of these cells is highly activated, which leads to this part of the patient’s PR. To the best of our knowledge, this finding has not been reported previously. Therefore, we reviewed the literature on the mechanism of sorafenib primary and acquired resistance and identified the characteristics of cell clusters that can antagonize the killing effect of sorafenib.

Chen et al. found that after treatment with sorafenib, the levels of c-Jun and p-c-Jun in HCC cells increased (Chen et al., 2016). Also, the expression level of *c-Jun* mRNA was inversely correlated with the efficacy of sorafenib. Therefore, we suggested that the activation of c-Jun may mediate acquired resistance and may exert a protective effect on sorafenib-induced cell growth inhibition. These observations are consistent with those of Hagiwara et al. (2012). The study showed a strong correlation between elevated JUN activity and PR to sorafenib in liver cancer. The current findings showed that JUN is not activated in all the tumor cells; only one cluster had this phenomenon. This is the first report on the role of JUN in the tumor microenvironment. In addition to the activation of JUN family genes, *ALDOA*, *EN O 1*, and *FOS* are also activated (Supplementary Figures S2D,F); these three transcription factors are the regulatory genes of the HIF pathway. Subsequently, we found that the HIF pathway in the PR cell cluster is activated and undergoing anaerobic metabolism, as detected by QuSAGE analysis. HIF and anaerobic metabolism play a critical role in acquired drug resistance. Several studies speculated that the number of blood vessels decreases gradually due to sorafenib administration, leading to hypoxia in the tissues and further activating HIF under such pressure (Wilson et al., 2014; Zhao et al., 2014; Won et al., 2015; Chen and Lou, 2017; Qiu et al., 2019). However, the current data demonstrated that JUN is activated in some cell clusters and undergoing anaerobic metabolism in tumor tissues without sorafenib. These tumor cells are likely to be differentiated from the liver bud hepatic cell. We speculated that due to the heterogeneity of the tumor, some cells exhibit a poor response to sorafenib directly. This cell cluster could also chemoattract other cells to differentiate into sorafenib-resistant clusters. In the PR-SA group, we observed that cluster five of cells might be effectuated towards differentiation into the PR group. A similar phenomenon was observed in the cluster four and cluster eight in the PR-SB, indicating that cell cluster with varied differentiation directions exists in tumor tissues (Supplementary Figures S1D,G).

The CellPhoneDB database analysis found that the three sorafenib PR cell clusters interacted through ALB and FcRn (Figure 4). Swiercz et al. demonstrated that tumor cells ingest a large amount of ALB through FcRn complex receptors to provide nutrients for proliferation or invasion (Swiercz et al., 2017). In addition, Dalloneau et al. found that FcRn expression is downregulated in patients with non-small cell lung cancer, which was related to the low survival rate of patients (Dalloneau et al., 2016). This finding was in agreement with that of Baker et al. (Baker et al., 2013). Similar to our findings, Howard et al. identifies overexpression of FcRn in several human cancer types with mechanistic data suggesting FcRn-driven albumin recruitment for increased cellular growth (Ju et al., 2016). Judith Blaine et al. and Paul A Gleeson et al. proved that FcRn is responsible for the recycling and transcytosis of albumin (Dylewski et al., 2019; Toh et al., 2019). As it pertains to the albumin homeostasis, FcRn deficient humans are hypoalbuminemic (Adonai et al., 2006). However, none of the studies have shown a correlation between FcRn and PR. Due to ALB is produced solely by hepatocytes (Pyzik et al., 2019), we infer that FcRn can more quickly transport ALB to liver cancer cells so that they can proliferate under the pressure of soranfenib. The metabolic environment of the cell line is different from the real microenvironment of the tumor *in vivo*. Nonetheless, based on the transcriptome sequencing data and results of *in vitro* experiments (Figure 6, Supplementary Figures S6), we suggest that the FcRn provides nutrients for the metabolism of the sorafenib-resistant cell line (huh-7) through uptake ALB also affects its overall proliferation and differentiation function; such a phenomenon was not observed in WT cell lines.

Due to the current status of patients’ medication and ethical requirements, it is impossible to use the tissues of liver cancer patients to study the mechanism of PR. Herein, we employed the PDX model to maximize the heterogeneity of tumor tissue. The results of scRNA-seq revealed that when some liver cancer patients were not treated with sorafenib, the JUN family genes in some liver cancer cells were over-activated, and the HIF pathway was metabolized through the high interaction between ALB and FcRn. Although the current results do not indicate that liver cancer cells affect the specific mechanism of *JUN* gene regulation by ALB binding to FcRn, our findings provide a new strategy to elucidate the mechanism underlying PR.

## Conclusion

Taken together, we identified a liver bud hepatic cell cluster associated with primary resistance to sorafenib in HCC PDX model. Our data demonstrate that the JUN transcription factor and HIF pathway were highly activated, and FcRn activation played a prominent role in primary resistance to Sorafenib. These results demonstrate that FcRn is a novel surface therapeutic oncotarget for anti-HCC agent combination with sorafenib.

## Data Availability

The original contributions presented in the study are publicly available. This data can be found here: GEO, accession GSE175716 (https://www.ncbi.nlm.nih.gov/geo/query/acc.cgi?acc=GSE175716)

## Abbreviation

HCC, hepatocellular carcinoma; PR, primary resistance; SA, sorafenib sensitive A; SB, sorafenib sensitive B; PDX, patient-derived xenograft; UMI, unique molecular identifier; SCENIC, single-cell regulatory network inference and clustering; QuSAGE, Quantitative Set Analysis for Gene Expression; BP, biological process; MF, molecular function; CC, cellular components.
