# Supplementary material for: Activation of FcRn Mediates a Primary Resistance Response to Sorafenib in Hepatocellular Carcinoma by Single-Cell RNA Sequencing
Source: Front Pharmacol. 2021 Aug 6;12:709343. doi: 10.3389/fphar.2021.709343 (PMC8379008; doi:10.3389/fphar.2021.709343)
Supplement: Supplementary file 1 [file Table1.DOCX]

Supplementary Material

Supplementary Table S1 Cell Type Table

|  | Cluster | Cell Type | Mark Gene | All Marker Gene | *P-*value |
| --- | --- | --- | --- | --- | --- |
| PR-SA | 0 | Liver bud hepatic cell | 35 | 812 | 0.00 |
|  |  | Regulatory T (Treg) cell | 49 | 812 | 0.00 |
|  | 2 | Liver bud hepatic cell | 25 | 336 | 0.00 |
|  |  | Regulatory T (Treg) cell | 28 | 336 | 0.00 |
|  |  | Exhausted CD8+ T cell | 13 | 336 | 0.05 |
|  | 3 | Liver bud hepatic cell | 20 | 256 | 0.00 |
|  |  | Regulatory T (Treg) cell | 16 | 256 | 0.03 |
|  | 4 | Liver bud hepatic cell | 24 | 445 | 0.00 |
|  |  | Regulatory T (Treg) cell | 28 | 445 | 0.01 |
|  | 6 | Liver bud hepatic cell | 24 | 284 | 0.00 |
|  |  | Regulatory T (Treg) cell | 21 | 284 | 0.00 |
|  |  | Exhausted CD8+ T cell | 14 | 284 | 0.01 |
|  | 7 | Exhausted CD4+ T cell | 10 | 493 | 0.03 |
|  | 9 | Liver bud hepatic cell | 22 | 351 | 0.00 |
|  |  | Regulatory T (Treg) cell | 24 | 351 | 0.00 |
| PR-SB | 0 | Liver bud hepatic cell | 28 | 415 | 0.00 |
|  |  | Regulatory T (Treg) cell | 29 | 415 | 0.00 |
|  | 1 | Regulatory T (Treg) cell | 48 | 659 | 0.00 |
|  |  | Liver bud hepatic cell | 27 | 659 | 0.00 |
|  | 2 | Exhausted CD8+ T cell | 10 | 140 | 0.00 |
|  |  | Regulatory T (Treg) cell | 13 | 140 | 0.00 |
|  | 3 | Liver bud hepatic cell | 26 | 515 | 0.00 |
|  |  | Regulatory T (Treg) cell | 31 | 515 | 0.01 |
|  | 5 | Liver bud hepatic cell | 24 | 359 | 0.00 |
|  |  | Regulatory T (Treg) cell | 24 | 359 | 0.01 |
|  | 6 | Liver bud hepatic cell | 26 | 688 | 0.00 |
|  |  | Regulatory T (Treg) cell | 37 | 688 | 0.02 |
|  | 7 | Regulatory T (Treg) cell | 32 | 588 | 0.02 |
