# Supplementary material for: Activation of FcRn Mediates a Primary Resistance Response to Sorafenib in Hepatocellular Carcinoma by Single-Cell RNA Sequencing
Source: Front Pharmacol. 2021 Aug 6;12:709343. doi: 10.3389/fphar.2021.709343 (PMC8379008; doi:10.3389/fphar.2021.709343)
Supplement: Supplementary file 2 [file Table2.DOCX]

Supplementary Material

Supplementary Table S2 Single Cell Gene Module 44 and 55 GO Analysis (BP) of PR-SA Group

| Module 44 GOTerm | *P-*Value | Enrichment | (-log_10_P) |
| --- | --- | --- | --- |
| regulation of protein deacetylation | 0.00 | 62.86 | 3.43 |
| DNA ligation | 0.00 | 31.43 | 2.77 |
| ubiquinone biosynthetic process | 0.00 | 22.86 | 2.48 |
| V(D)J recombination | 0.00 | 20.95 | 2.41 |
| regulation of protein ubiquitination | 0.00 | 19.34 | 2.34 |
| chromatin modification | 0.00 | 3.37 | 2.31 |
| mRNA transport | 0.01 | 5.78 | 2.29 |
| positive regulation of potassium ion transmembrane transport | 0.01 | 17.96 | 2.27 |
| ceramide metabolic process | 0.01 | 16.76 | 2.21 |
| cell cycle | 0.01 | 2.40 | 2.21 |
| cellular response to heat | 0.01 | 5.35 | 2.17 |
| negative regulation of the force of heart contraction | 0.01 | 125.73 | 2.10 |
| transport of viral material towards nucleus | 0.01 | 125.73 | 2.10 |
| positive regulation of DNA damage checkpoint | 0.01 | 125.73 | 2.10 |
| glycine receptor clustering | 0.01 | 125.73 | 2.10 |
| Module 55 GOTerm | P-Value | Enrichment | (-log10P) |
| translational elongation | 0.00 | 13.42 | 7.66 |
| translation | 0.00 | 5.72 | 5.47 |
| RNA metabolic process | 0.00 | 5.70 | 4.09 |
| gene expression | 0.00 | 3.06 | 4.00 |
| translational termination | 0.00 | 10.23 | 3.89 |
| mRNA metabolic process | 0.00 | 5.58 | 3.59 |
| SRP-dependent cotranslational protein targeting to membrane | 0.00 | 8.40 | 3.49 |
| viral transcription | 0.00 | 8.31 | 3.47 |
| nuclear-transcribed mRNA catabolic process, nonsense-mediated decay | 0.00 | 7.60 | 3.29 |
| protein localization to mitochondrion | 0.00 | 45.58 | 3.10 |
| viral life cycle | 0.00 | 6.28 | 2.92 |
| toxin transport | 0.00 | 13.67 | 2.87 |
| translational initiation | 0.00 | 5.74 | 2.74 |
| regulation of autophagy | 0.00 | 11.67 | 2.67 |
| cellular protein metabolic process | 0.00 | 2.73 | 2.45 |
