# Supplementary material for: Activation of FcRn Mediates a Primary Resistance Response to Sorafenib in Hepatocellular Carcinoma by Single-Cell RNA Sequencing
Source: Front Pharmacol. 2021 Aug 6;12:709343. doi: 10.3389/fphar.2021.709343 (PMC8379008; doi:10.3389/fphar.2021.709343)
Supplement: Supplementary file 3 [file Table3.DOCX]

Supplementary Material

Supplementary Table S3 Single Cell Gene Module 7 and 22 GO Analysis (BP) of PR-SB Group

| Module 7 GOTerm | *P*-Value | Enrichment | (-log_10_P) |
| --- | --- | --- | --- |
| cell cycle | 0.00 | 9.83 | 57.17 |
| mitotic nuclear division | 0.00 | 16.39 | 55.24 |
| cell division | 0.00 | 12.46 | 49.49 |
| mitotic cell cycle | 0.00 | 11.34 | 48.59 |
| chromosome segregation | 0.00 | 22.53 | 23.63 |
| mitotic sister chromatid segregation | 0.00 | 35.84 | 13.43 |
| CENP-A containing nucleosome assembly | 0.00 | 22.63 | 13.11 |
| G2/M transition of mitotic cell cycle | 0.00 | 9.70 | 12.43 |
| small GTPase mediated signal transduction | 0.00 | 3.66 | 10.35 |
| DNA replication | 0.00 | 8.19 | 9.97 |
| chromosome condensation | 0.00 | 35.84 | 9.53 |
| G1/S transition of mitotic cell cycle | 0.00 | 7.57 | 8.89 |
| nucleosome assembly | 0.00 | 8.29 | 8.86 |
| cellular response to DNA damage stimulus | 0.00 | 4.34 | 8.82 |
| DNA repair | 0.00 | 4.57 | 8.53 |
| Module 22 GOTerm | P-Value | Enrichment | (-log10P) |
| mitotic cell cycle | 0.00 | 5.10 | 8.25 |
| mitotic cytokinesis | 0.00 | 26.68 | 7.15 |
| cell cycle | 0.00 | 3.89 | 7.04 |
| mitotic nuclear division | 0.00 | 5.38 | 6.04 |
| cell division | 0.00 | 4.56 | 5.98 |
| microtubule-based movement | 0.00 | 9.15 | 4.31 |
| cellular response to DNA damage stimulus | 0.00 | 3.50 | 4.05 |
| cell proliferation | 0.00 | 3.61 | 3.89 |
| mitotic spindle assembly checkpoint | 0.00 | 13.34 | 3.66 |
| G2/M transition of mitotic cell cycle | 0.00 | 5.62 | 3.60 |
| regulation of cell cycle | 0.00 | 5.22 | 3.40 |
| CENP-A containing nucleosome assembly | 0.00 | 11.24 | 3.37 |
| chromosome segregation | 0.00 | 7.62 | 3.30 |
| mitotic sister chromatid segregation | 0.00 | 16.01 | 3.09 |
| microtubule bundle formation | 0.00 | 16.01 | 3.09 |
